# A Cellular Model of Amyotrophic Lateral Sclerosis to Study the Therapeutic Effects of Extracellular Vesicles from Adipose Mesenchymal Stem Cells on Microglial Activation

**DOI:** 10.3390/ijms25115707

**Published:** 2024-05-24

**Authors:** Sylwia Dabrowska, Ermanna Turano, Ilaria Scambi, Federica Virla, Alice Nodari, Francesco Pezzini, Mirco Galiè, Bruno Bonetti, Raffaella Mariotti

**Affiliations:** 1 Department of Neurosciences, Biomedicine and Movement Sciences, University of Verona, Strada Le Grazie 8, 37134 Verona, Italy; sdabrowska@imdik.pan.pl (S.D.); ermanna.turano@univr.it (E.T.); ilaria.scambi@univr.it (I.S.); federica.virla@univr.it (F.V.); alice.nodari@univr.it (A.N.); mirco.galie@univr.it (M.G.); 2 NeuroRepair Department, Mossakowski Medical Research Institute, Polish Academy of Sciences, Pawinskiego Street 5, 02-106 Warsaw, Poland; 3 Department of Surgery, Dentistry, Paediatrics and Gynaecology (Child Neurology and Psychiatry), University of Verona, 37134 Verona, Italy; francesco.pezzini@univr.it; 4 Neurology Unit, Azienda Ospedaliera Universitaria Integrata, 37126 Verona, Italy; bruno.bonetti@univr.it

**Keywords:** microglial cells, SOD1(G93A), extracellular vesicles, amyotrophic lateral sclerosis, SIM-A9, stem cells, exosomes

## Abstract

Amyotrophic lateral sclerosis (ALS) is a fatal neurodegenerative disease characterized by the progressive degeneration of upper and lower motor neurons (MNs) in the brain and spinal cord, leading to progressive paralysis and death. Increasing evidence indicates that neuroinflammation plays an important role in ALS’s pathogenesis and disease progression. Neuroinflammatory responses, primarily driven by activated microglia and astrocytes, and followed by infiltrating peripheral immune cells, contribute to exacerbate/accelerate MN death. In particular, the role of the microglia in ALS remains unclear, partly due to the lack of experimental models that can fully recapitulate the complexity of ALS’s pathology. In this study, we developed and characterized a microglial cell line, SIM-A9-expressing human mutant protein Cu^+^/Zn^+^ superoxide dismutase_1 (SIM-A9hSOD1(G93A)), as a suitable model in vitro mimicking the microglia activity in ALS. The expression of hSOD1(G93A) in SIM-A9 cells induced a change in their metabolic activity, causing polarization into a pro-inflammatory phenotype and enhancing reactive oxygen species production, which is known to activate cell death processes and apoptosis. Afterward, we used our microglial model as an experimental set-up to investigate the therapeutic action of extracellular vesicles isolated from adipose mesenchymal stem cells (ASC-EVs). ASC-EVs represent a promising therapeutic treatment for ALS due to their neuroprotective and immunomodulatory properties. Here, we demonstrated that treatment with ASC-EVs is able to modulate activated ALS microglia, reducing their metabolic activity and polarizing their phenotype toward an anti-inflammatory one through a mechanism of reduction of reactive oxygen species.

## 1. Introduction

Amyotrophic lateral sclerosis (ALS) is a fatal neurodegenerative disease characterized by the loss of upper and lower motor neurons in the motor cortex, brainstem and spinal cord, leading to progressive muscle paralysis. The disease is familial in 5–10% of cases, and 15% of the familial cases are attributable to mutations in the gene encoding Cu^+^/Zn^+^ superoxide dismutase (SOD1), but some studies have demonstrated that SOD1 dysfunction could also have a pathogenic role in sporadic ALS [1,2]. Unfortunately, no effective therapies exist that could significantly extend and improve the quality of life of people with ALS. Consequently, most patients die within 2–5 years from the first disease symptoms [3]. The pathogenesis of ALS is very complex and involves many different cellular mechanisms, such as oxidative stress, mitochondrial dysfunction, glutamate excitotoxicity, axonal transport, impaired protein degradation, hypermetabolism and RNA defects. Recently, the role of neuroinflammation in ALS’s pathogenesis was also emphasized [4,5,6].

Several studies support the idea that activated microglia play a crucial role in the disease course of ALS [7,8,9]. In particular, microglial cells were found to be highly activated in the murine model of ALS, and closely related to the severity of motor neuron degradation in ALS [10]. The microglial cells show a broad spectrum of activation that includes two intermediate phenotypes, M1—pro-inflammatory and M2—anti-inflammatory, playing a toxic or protective role in ALS’s pathogenesis, respectively [11,12,13]. These two microglial phenotypes can pass into each other in different contexts, contributing to the pathogenesis of the disease [14]. Several studies reported that microglia exhibit the M2 anti-inflammatory phenotype in the early stages of the disease and progressively shift toward the classically activated M1 phenotype [15]. Microglial cell activation contributes to the death of motor neurons due to the secretion of neurotoxic factors that accelerate the progression of the disease [16].

Together with inflammation, oxidative stress and mitochondrial dysfunction play a decisive role in the pathogenesis of ALS [17]. Reactive oxygen species (ROS) have been shown to play a crucial role in motor neuron death [18,19] and ROS biomarkers have been identified in the brain tissue, cerebrospinal fluid, blood and urine of ALS patients [20].

Thus, the modulation of microglial activity and of ROS release may be a potential target for elaborating effective ALS therapy to cure people with ALS.

The use of non-pharmacological therapies has become a possible innovative treatment for ALS. In particular, the use of mesenchymal stem cells (MSCs) has proved to be a valid approach [21,22] due to their self-renewal ability, differentiation potential, and immunomodulatory and neuroprotective properties [23,24]. The therapeutic potential of MSCs is at least partly related to the release of extracellular vesicles (EVs), which express similar therapeutic properties as their cells of origin [25]. EVs have protective and regenerative capacities and the ability to modulate the immune response [25,26,27], and they could represent an alternative to cell-based therapy [28,29,30]. In fact, the neuroprotective effect of EVs derived from mesenchymal stem cells has been extensively reported in the literature in several models of neurodegeneration [31,32,33,34,35]. In particular, our group demonstrated that MSC-EVs protect motor neurons from degeneration in ALS models, both in vitro and in vivo [36], and are able to rescue mitochondrial function in NSC-34 motor neurons [36,37]. However, the effect of EVs on microglia cells in ALS has not been well investigated. In the present study, we elaborated a cellular model stably transfecting the microglial SIM-A9 cell line [38] with the human SOD1(G93A) gene to evaluate the impact of MSC-EVs on an in vitro model of ALS microglia.

## 2. Results

### 2.1. Doxycycline Induces the Expression of hSOD1(G93A) in Microglial Cells

**Figure 1:**
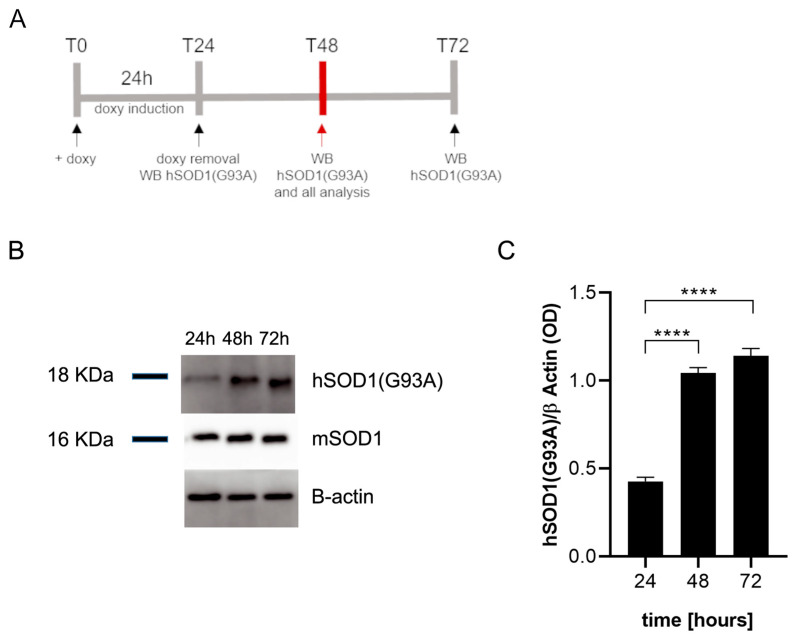
Western blot analysis of SIM-A9hSOD1(G93A) microglial cells. (**A**) Schematic illustration of the experimental timeline: T0, doxycycline has been added to the culture medium; T24, doxycycline has been removed from the culture medium and hSOD1(G93A) was detected; T48 hSOD1(G93A) was detected 24 h after doxycycline removal, T72 hSOD1(G93A) was detected 48 h after doxycycline removal. (**B**) The representative Western blot performed on microglial cells to detect the expression of hSOD1(G93A) at 24, 48, and 72 h after doxycycline (doxy) addition. (**C**) The semi-quantitative analysis of hSOD1(G93A) expression revealed high levels of protein after the incubation with doxycycline. Data are shown as mean ± SEM; one-way ANOVA with Tukey’s post hoc correction; **** *p* < 0.0001.

We first verified that doxycycline induces the expression of the hSOD1(G93A) protein in transfected SIM-A9 cells by Western blot analysis according to the timeline reported in Figure 1A. The results revealed the presence of the hSOD1(G93A) protein in microglial cells, expressed as optical density (OD) values, already 24 h after doxycycline addition to the culture media. The expression of the hSOD1(G93A) protein increased significantly 48 h (*p* < 0.0001) and 72 h (*p* < 0.0001) after T0 (Figure 1B). No differences were observed between 48 and 72 h in the amount of hSOD1(G93A), suggesting a plateau was reached in terms of protein expression (Figure 1C). All the experiments were performed at T48 (*n* = 3 replicates for each condition). The presence of the hSOD1(G93A) protein was also confirmed by immunofluorescence staining in SIM-A9hSOD1(G93A) microglial cells following 48 h of doxycycline treatment (Supplementary Figure S1).

### 2.2. hSOD1(G93A) Protein Is Able to Induce Microglial Activation

**Figure 2:**
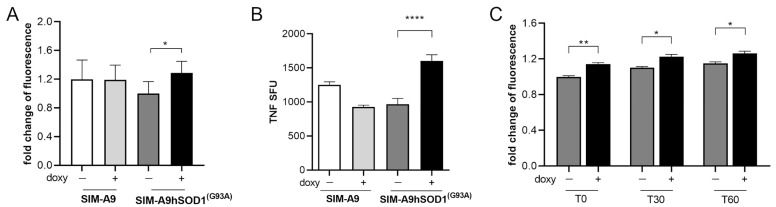
Evaluation of SIM-A9hSOD1(G93A) microglial cells activation. (**A**) WST assay on SIM-A9 and SIM-A9hSOD1(G93A) cells treated (doxy+) or untreated (doxy−) with doxycycline (5 μg/mL). (**B**) ELISpot detection of TNF-α release in microglial SIM-A9 naïve and SIM-A9hSOD1(G93A) treated (doxy+) or untreated (doxy−) with doxycycline. The TNF-α quantification was expressed as spot-forming units (SFU). Significant differences indicated as * *p* < 0.05 and **** *p* < 0.0001 were analyzed via two-way ANOVA with Tukey’s post hoc correction. Data are shown as mean ± SEM. (**C**) ROS production by SIM-A9hSOD1(G93A) cells, untreated (doxy−) or treated (doxy+) with doxycycline at different incubation times with the CM-H2DCFDA dye. Significant differences indicated as * *p* < 0.05 and ** *p* < 0.01 were assessed via two-way ANOVA with Tukey’s post hoc correction. Data are shown as mean ± SEM.

Microglial naïve (SIM-A9) and SIM-A9hSOD1(G93A) cells were incubated with 5 μg/mL of doxycycline for 24 h to induce the transcription of hSOD1(G93A), as previously established [39]. Then, a WST assay was performed to verify the metabolic activation of microglial cells 24 h after the removal of doxycycline (T48) (*n* = 3 replicates for each condition). No statistical difference was observed between SIM-A9 cells exposed or not to doxycycline, indicating that the presence of doxycycline is not able to modify the metabolic activation of SIM-A9 cells (Figure 2A). Conversely, the expression of hSOD1(G93A) following doxycycline treatment induces a significant increase in SIM-A9hSOD1(G93A) metabolic activity compared to SIM-A9hSOD1(G93A) cells that did not undergo the doxycycline treatment (*p* = 0.00258) (Figure 2A).

Noteworthily, we revealed increased TNF-α release by doxycycline-treated SIM-A9hSOD1(G93A) cells compared to untreated SIM-A9hSOD1(G93A) cells and SIM-A9 naïve cells (Figure 2B) (*p* < 0.0001) (*n* = 3 replicates for each condition).

We then evaluated the release of reactive oxygen species (ROS) by SIM-A9hSOD1(G93A) cells, which is known to be one of the cellular mechanisms implicated in the pathogenesis of ALS. An ROS-sensitive probe (CM-H2DCFDA) was used to analyze ROS production by the SIM-A9hSOD1(G93A) cells (*n* = 6 replicates for each condition). Our results showed that doxycycline-treated SIM-A9hSOD1(G93A) cells displayed higher levels of ROS compared to untreated SIM-A9hSOD1(G93A) cells. In particular, we found increased fluorescence intensity of the ROS-sensitive probe (CM-H2DCFDA) immediately after incubation with the dye (T0 *p* = 0.0011), as well as after 30 (T30 *p* = 0.03401) and 60 min (T60 *p* = 0.0280; Figure 2C). These results indicate that the induction of the hSOD1(G93A) gene by doxycycline enhanced the ROS production by SIM-A9hSOD1(G93A) cells.

### 2.3. hSOD1(G93A) Expression Reveals Morphological Changes Phenotype in Microglial Cells

**Figure 3:**
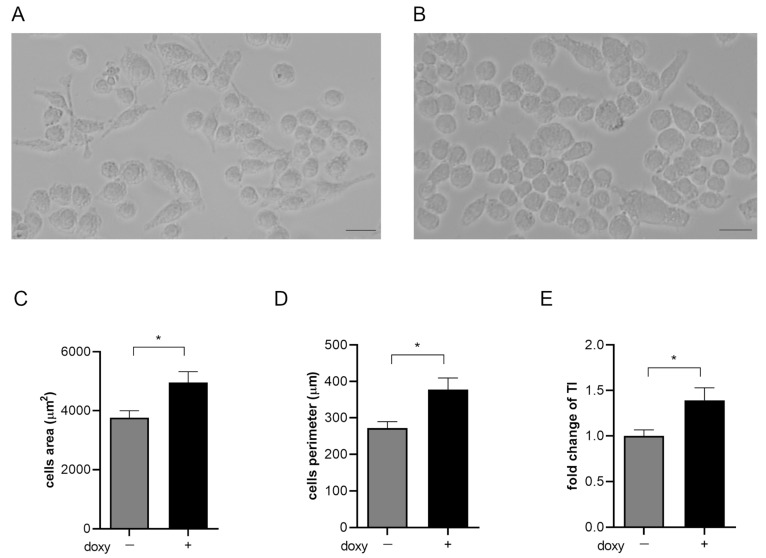
Morphological analysis of SIM-A9hSOD1(G93A) microglial cells. Representative images of untreated microglial SIM-A9hSOD1(G93A) cells (**A**) and following incubation with doxycycline (doxy+) (**B**). Graphs show the quantitative analysis of cells’ morphology parameters, namely cell body area (3757.53 ± 717.21 vs. 4961.03 ± 1099.24 μm^2^) (**C**), cell perimeter (272.15 ± 51.33 vs. 377.68 ± 96.67 μm) (**D**) and transformation index (1.572 ± 0.31 vs. 2.318 ± 0.8) (**E**). Magnification 10×, scale bar 100 μm. Significant differences indicated as * *p* < 0.05 were assessed via the Student’s T test. Data are shown as mean ± SEM.

The microglial SIM-A9hSOD1(G93A) cells assumed different morphology following the induction with doxycycline. We have observed that untreated SIM-A9hSOD1(G93A) cells appeared elongated with long projections compared to SIM-A9hSOD1(G93A) treated with doxycycline, which were larger and with shorter projections. The expression of the hSOD1(G93A) construct seemed to be able to modify the cell shape, transforming it into an amoeboid-like form (T48) (Figure 3A,B). Moreover, morphological analysis confirmed that this modification was accompanied by a significant increase in the cell area (24.27%; *p* = 0.0142) (Figure 3C), elongation of the cell perimeter (27.94%; *p* = 0.0106) (Figure 3D), and an increment of the transformation index (32.18%; *p* = 0.0189) in SIM-A9hSOD1(G93A) after doxycycline induction (Figure 3E) (*n* = 3 replicates for each condition).

### 2.4. hSOD1(G93A) Expression Triggers a Detrimental Phenotype in Microglial Cells

**Figure 4:**
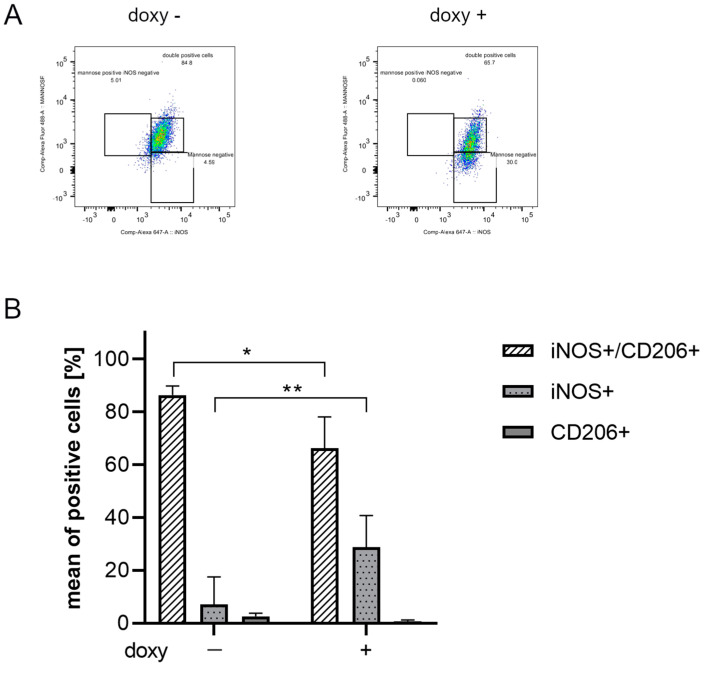
Evaluation of an inflammatory phenotype in SIM-A9hSOD1(G93A) microglial cells. (**A**) Flow cytometry representative plots of untreated (doxy-, left plot) and doxycycline-treated (doxy+, right plot) SIM-A9hSOD1(G93A) cells, labelled for INOS and CD206. (**B**) Quantitative flow cytometry analysis of SIM-A9hSOD1(G93A) cells, positive for INOS (INOS+), CD206 (CD206+) or for both markers (INOS+/CD206+). The results are represented as a percentage of the mean of positive cells. Significant differences indicated as * *p* < 0.05 and ** *p* < 0.01 were assessed via two-way ANOVA with Tukey’s post hoc correction. Data are shown as mean ± SEM.

Flow cytometry analysis was performed to verify if hSOD1(G93A) expression could promote a pro-inflammatory phenotype in microglial cells. In particular, the inflammatory phenotype was ascertained in SIM-A9hSOD1(G93A) cells in terms of the expression of M1 pro-inflammatory (INOS) or M2 anti-inflammatory (CD206 Mannose Receptor-positive) markers (*n* = 3 replicates for each condition). The analysis revealed that SIM-A9hSOD1(G93A) cells, in the absence of doxycycline treatment, do not show a specific M1 or M2 phenotype, with the majority remaining undifferentiated (86.27%). Indeed, the percentages of INOS-positive and CD206-positive cells remained very low (7.13% and 2.65%, respectively) (Figure 4A,B). Interestingly, doxycycline treatment of SIM-A9hSOD1(G93A) cells induced a significant increase in INOS-positive compared to CD-206 positive cells (18.11% and 0.81%, respectively) and a reduction in undifferentiated cells (76.75%) (Figure 4A,B). Thus, the presence of the hSOD1(G93A) protein was able to reduce the undifferentiated phenotype (*p* = 0.0180; * *p* < 0.05) and to drive SIM-A9hSOD1(G93A) cells versus an M1 pro-inflammatory phenotype (*p* = 0.0091; ** *p* < 0.01) (Figure 4B), also observed and quantified by immunofluorescence staining (Supplementary Figure S2).

### 2.5. Characterization of ASC-EVs

To characterize the isolated ASC-EVs, morphological and quantitative parameters were evaluated. NTA showed measurements of size and concentration for ASC-EVs consistent with the data reported in the literature [40] (Supplementary Figure S3A). The data are reported in Table 1 (*n* = 3 replicates for each condition). The ultrastructural analysis of the ASC-EVs performed by transmission electron microscopy (TEM) confirmed the proper isolation of round-shaped vesicles with lipid bilayers, from 50 to 150 nm in diameter (mean = 130.9 ± 52.1 nm) (Supplementary Figure S3B). The expression of specific EV markers was assessed by Western blot (Supplementary Figure S3C). Altogether, these results proved that the size, morphology, and presence of specific markers were consistent with the proper isolation of extracellular vesicles from adipose tissue mesenchymal stem cells (Supplementary Figure S3A–C).

### 2.6. ASC-EVs Are Able to Reduce the Activation of Microglial Cells

Since SIM-A9hSOD1(G93A) cells treated with doxycycline are a reliable model of activated/detrimental ALS microglia, we further examined the capability of ASC-EVs to modulate microglial activation.

**Figure 5:**
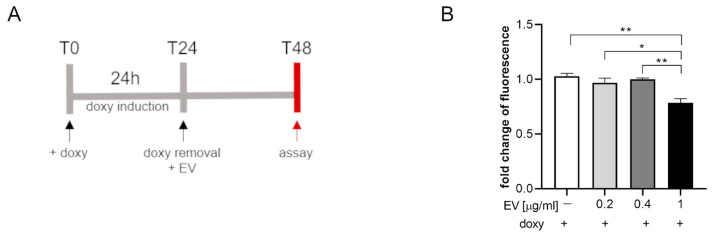
The WST assay on SIM-A9hSOD1(G93A) cells treated with ASC-EV. (**A**) Experimental timeline of SIM-A9hSOD1(G93A) cells’ treatment with ASC-EV. (**B**) Metabolic activity of microglial SIM-A9hSOD1(G93A) cells treated with 0, 0.2, 0.4, and 1 µg/mL of ASC-EV for 24 h. Significant differences indicated as * *p* < 0.05 and ** *p* < 0.01 were assessed via one-way ANOVA with Tukey’s post hoc correction. Data are shown as mean ± SEM.

SIM-A9hSOD1(G93A) cells were incubated with different concentrations of ASC-EVs (0.2–0.4–1 µg/mL) for 24 h (Figure 5A). The WST assay showed a significant decrease in fluorescence in SIM-A9hSOD1(G93A) cells incubated with ASC-EVs (1 µg/mL) compared to cells not exposed to EVs (*p* = 0.0021) (Figure 5B) (*n* = 3 replicates for each condition). No significant effects were observed in terms of the ASC-EVs in reducing the release of TNF-α by SIM-A9hSOD1(G93A) cells.

### 2.7. ASC-EVs Are Able to Modulate the Phenotype of Microglial Cells

**Figure 6:**
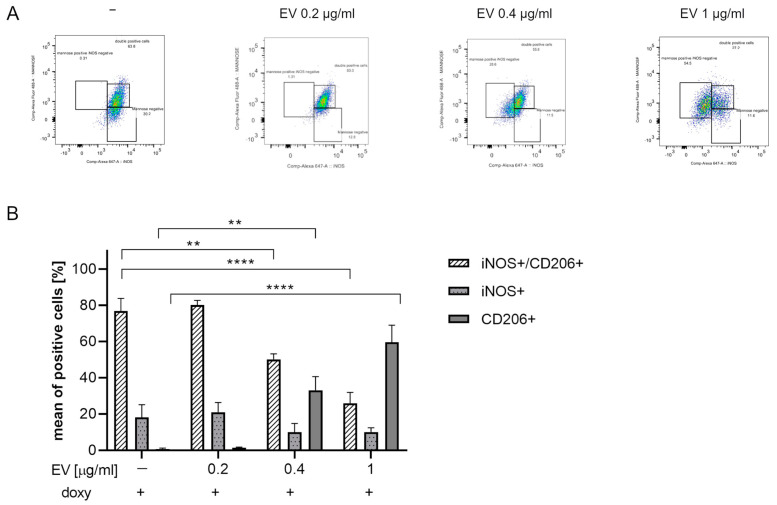
Evaluation of ASC-EVs’ capability to modulate SIM-A9hSOD1(G93A) cells’ phenotype. (**A**) Flow cytometry representative plots of SIM-A9hSOD1(G93A) cells labelled for INOS and CD206 markers after the treatment with 0.2, 0.4 and 1 µg/mL of ASC-EVs (from left to right). INOS+, CD206+ and INOS+/CD206+ cells were highlighted in three squares. (**B**) Quantitative flow cytometry analysis of microglia SIM-A9hSOD1(G93A) cells after treatment with different concentrations of ASC-EVs. Significant differences indicated as ** *p* < 0.01 and **** *p* < 0.0001 were assessed by two-way ANOVA with Tukey’s post hoc correction. Data are shown as mean ± SEM.

We then assessed whether ASC-EVs can induce phenotypical changes in SIM-A9hSOD1(G93A) cells. For this purpose, the expression of INOS and anti-CD206 markers was evaluated by flow cytometry analysis after ASC-EV treatment (Figure 6A).

The percentage of INOS+/CD206+ double-positive cells significantly decreased in a dose-dependent manner and in particular after incubation with 1 µg/mL ASC-EVs (*p* < 0.0001). Interestingly, a concomitant increase in the percentage of CD206+ cells (*p* < 0.0001) was detected (Figure 6B) (*n* = 3 replicates for each condition).

### 2.8. ASC-EVs Are Able to Reduce ROS Release by Microglial Cells

**Figure 7:**
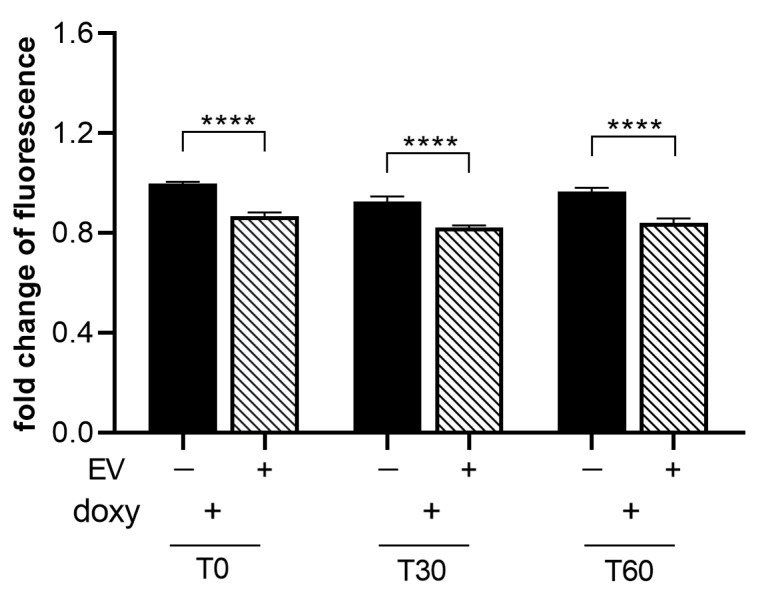
Evaluation of ASC-EVs’ capability to reduce ROS release by SIM-A9hSOD1(G93A) cells. ROS production in SIM-A9hSOD1(G93A) cells after treatment with 1 ug/mL of ASC-EVs at different incubation times with the CM-H2DCFDA dye. Significant differences indicated as **** *p* < 0.0001 were assessed by two-way ANOVA with Tukey’s post hoc correction. Data are shown as mean ± SEM.

To assess the effect of ASC-EVs in modulating ROS release by activated SIM-A9hSOD1(G93A) cells, we treated microglial cells with 1 µg/mL ASC-EVs (the concentration observed to be more effective in inducing phenotypical changes in SIM-A9hSOD1(G93A) cells) (*n* = 6 replicates for each condition). We observed a ~15% reduction in CMH2DCFDA fluorescence intensity at different time points after incubation with the dye, indicating that the ASC-EV treatment was able to significantly decrease the production of ROS in SIM-A9hSOD1(G93A) cells (Figure 7).

## 3. Discussion

ALS is a neurodegenerative disease characterized by progressive and irreversible degeneration of both upper and lower motor neurons in the motor cortex, brainstem and spinal cord [41]. Although motor neurons are primarily affected in ALS, a key role in the disease progression is played by non-neuronal cells [42]. Several studies have emphasized the effect of glial cells on motor neuron survival. Indeed, experiments focused on familial ALS and SOD1 mutations highlighted the increase in motor neuron mortality in the presence of reactive astrocytes [43] and microglia [44,45]. Previous studies by Lee and co-workers showed that replacing SOD1 microglia with healthy microglia slowed the disease progression and prolonged the survival of mice in a murine model of ALS [46]. It was revealed that microglial cells release pro-inflammatory cytokines and neurotoxic factors, causing motor neurons’ death in the motor cortex and disease progression in a wobbler mouse ALS model [47]. In this regard, it is essential to investigate and deepen the role of the microglia and its involvement in the pathogenesis of ALS.

The use of models in vitro to understand specific cellular mechanisms in ALS appears to be very useful, since they provide a controlled environment with low variability and high repeatability, and they are easy to culture and enable the study of distinct pathogenic mechanisms of disease, thus representing an efficient alternative to primary culture [38] and limiting the use of animals [48].

Here, we show a novel cellular model of ALS microglia using an immortalized microglial SIM-A9 cell line stably transfected with hSOD1 gene carrying the G93A mutation. The obtained cell line, SIM-A9hSOD1(G93A), showed a “detrimental” phenotype characterized by mildly increased metabolic activity, together with a significant release of TNF-α, a major mediator of inflammatory response [49,50]. As observed by Massenzio and co-workers, intracellular SOD1 accumulation leads to microglial cell activation and neurotoxicity [51]. Noteworthily, we revealed increased ROS production in SIM-A9hSOD1(G93A) cells. Indeed, it has been demonstrated that misfolded mutated SOD1 proteins aggregate, causing the accumulation of ROS [19]. In addition, murine models of ALS have shown the involvement of ROS in the early stage of ALS [52,53]. In line with our data, the rapid increase in ROS release in SIM-A9hSOD1(G93A) cells suggests that the expression of the mutated SOD1 protein could be an early signal to commutate the microglia versus a harmful phenotype.

Furthermore, the microglial activation in SIM-A9hSOD1(G93A) is accompanied by a change in cellular morphology, with a transition from a ramified to an amoeboid-like shape, generally representing an intermediate activation state and a fully activated and phagocytic microglia, respectively [54,55], together with an increase in the cells’ area, perimeter and transformation index. Similar morphological changes were observed by Gill and collaborators in microglia cells after treatment with lipopolysaccharide [56].

Moreover, following exposure to doxycycline, we observed an increasing percentage of SIM-A9hSOD1(G93A) INOS+ cells with a concomitant decrease in CD206+ cells. These markers are commonly used to identify, respectively, the phenotype of “M1 microglia”, which induces inflammation and neurotoxicity, or “M2 microglia”, which promotes an anti-inflammatory phenotype supporting healing [57]. These two phenotypes are dynamic and can change in a continuum of different intermediate phenotypes [14,58]. Indeed, transcriptomic studies have shown that the M1 and M2 states represent a spectrum of activation patterns rather than separate cell subtypes [59]. Therefore, microglial cells can switch from one phenotype to another, depending on the milieu or the factors by which they are stimulated [59,60,61].

In this regard, our experiments revealed that a high percentage of SIM-A9hSOD1(G93A) cells simultaneously express both M1 and M2 markers, suggesting that the majority of cells are not polarized in a specific phenotype. Similar observations have been reported in other studies. These studies emphasize the heterogeneous nature of microglial polarization, suggesting that microglia could exhibit many activation states and express both M1 and M2 markers rather than being entirely polarized into the M1 pro-inflammatory or M2 anti-inflammatory phenotype [62,63,64,65].

However, the presence of SOD1(G93A)-induced metabolic activation, release of TNF-α and ROS, morphological changes and increased expression of M1 markers suggest a transition toward a “detrimental” microglia phenotype.

We then focused our attention on the therapeutic effect of ASC-EVs on microglial SIM-A9hSOD1(G93A) cells. Extracellular vesicles display immunomodulatory properties in different disease models in vitro and in vivo [66,67,68]. It has been demonstrated that EVs can inhibit microglial cell activation and promote a pro-inflammatory phenotype in different models [67,69,70,71,72]. Our study showed that the treatment of microglial SIM-A9hSOD1(G93A) cells with 1 µg/mL of ASC-EVs for 24 h significantly decreased the metabolic activity of microglial cells, whereas lower concentrations had no effect. These results indicate that the application of high doses of ASC-EVs is essential to reduce the metabolic activity of microglial SOD1 cells in vitro, with a reduction in INOS+ cells and a concomitant increase in CD206+ cells. These findings are in agreement with a study that revealed that MSC-EVs promoted the polarization of microglial cells by suppressing INOS expression and enhancing CD206 and Arginase-1 expression in a model of traumatic brain injury [73]. Similarly, the administration of the antioxidant minocycline was observed to delay the pathogenesis of mSOD1G93A mice by selectively attenuating the induction of M1 microglia markers during the progressive phase, without affecting the transient enhancement of the expression of M2 microglia markers at the early-onset stage [74].

Noteworthily, ASC-EV treatment reduced ROS release by SIM-A9hSOD1(G93A) cells, underlying their capacity to counteract the oxidative effects induced by mutated SOD1 expression, as previously demonstrated in a different experimental setting [75]. Accordingly, we recently demonstrated the efficiency of ASC-EVs in rescuing the function of mitochondria in NSC-34 motor neuron cells overexpressing the SOD1(G93A) mutation [39]. Altogether, these findings suggest the dual action of ASC-EVs to modulate mitochondrial dysfunction and to reduce the ROS production by microglial cells and the oxidative stress. Although these results are promising, the present study suffers from a few limitations. Our cellular model will not fully capture the complexity of the in vivo system. In particular, the application of the model overexpressing hSOD1(G93A) does not fully capture ALS’s pathology, which involves different genetic mutations and multiple pathogenic mechanisms. However, SOD1 models are among the most frequently used systems to investigate ALS disease. Finally, in view of the future clinical application of EVs, the establishment of several parameters (optimal EV doses, time of EV administration, delivery route and the safety profile) may be challenging for potential ALS therapy.

Our findings support ASC-EVs as a therapeutic strategy for the treatment of ALS, not only for their neuroprotective action directly on motor neurons but also for modulating microglial activation and contrasting oxidative stress.

## 4. Materials and Methods

### 4.1. Cell Cultures 

The microglial SIM-A9 cell line was purchased from ABM (Richmond, BC, Canada). Microglial SIM-A9 naïve cells were stably transfected with the human SOD1 gene (hSOD1) carrying the G93A mutation, SIM-A9hSOD1(G93A), to set up an in vitro microglial model of ALS. The hSOD1(G93A) gene expression was under the control of a doxycycline-inducible promoter [37]. Microglial SIM-A9 cells and SIM-A9hSOD1(G93A) cells were cultured in DMEM-F12 (GIBCO Life Technologies, Thermo Fisher Scientifics, Carlsbad, CA, USA) supplemented with 10% heat-inactivated fetal bovine serum (FBS; GIBCO, Thermo Fisher Scientifics, Carlsbad, CA, USA), 100 U/mL penicillin, and 100 μg/mL streptomycin (GIBCO, Thermo Fisher Scientifics, Carlsbad, CA, USA) at 37 °C in 5% CO_2_. For the experiments, we used SIM-A9 microglial naïve cells and SIM-A9hSOD1(G93A) cells treated or not with 5 μg/mL doxycycline (Doxy, Clontech, Mountain View, CA, USA) for 24 h. Microglial SIM-A9 and SIM-A9hSOD1(G93A) cells not treated with doxycycline were used as a control.

### 4.2. Western Blot

Western blot was performed to assess the SOD expression in SIM-A9hSOD1(G93A) following 24, 48 and 72 h of doxycycline induction. Microglial cells were lysed for 20 min on ice in RIPA buffer supplemented with protease inhibitors (Thermo Fisher Scientifics, Carlsbad, CA, USA) and the protein concentration was quantified using a Bradford assay (Sigma Aldrich, Saint Louis, MO, USA). The samples were denatured by boiling at 95 °C in Laemmli SDS sample buffer, separated by 12% SDS polyacrylamide gel electrophoresis, and transferred to nitrocellulose membrane. Then, the membranes were blocked with TBST buffer (10 mM Tris–HCl, 100 mM NaCl, 0.1% (*v*/*v*) Tween 20, pH 7.5) and 5% milk for 1 h at room temperature. The primary antibodies were added overnight at 4 °C as follows: anti-His-tag (α-His) (Aviva Systems Biology, San Diego, CA, USA; 1:1000) to recognize the epitope of 6× His-tag encoded by the human SOD1(G93A) (hSOD1(G93A)), anti-murine SOD1 (mSOD1: Novus Biologicals, Littleton, CO, USA; 1:1000), and anti-ß-actin (Santa Cruz Biotechnology, Santa Cruz, CA, USA; 1:5000) was used as an internal control. Then, the membranes were incubated with the appropriate HRP-conjugated secondary antibodies. The membranes were developed using Pierce ECL Plus from Thermo Fisher Scientific (Rockford, IL, USA). The experiment was performed in triplicate for each condition.

### 4.3. ASC-EV Isolation and Characterization

The adipose mesenchymal stem cells (ASCs) were isolated from the inguinal adipose tissue of 8/12 week-old C57BL/6 mice (Charles River, Milan, Italy), as previously described [37]. The inguinal fat was incubated in Hank’s Balanced Salt Solution (HBSS, Thermo Fisher Scientifics, Carlsbad, CA, USA) with collagenase type I (Thermo Fisher Scientifics, Carlsbad, CA, USA) and BSA (AppliChem, Darmstadt, Germany) and then centrifuged to obtain a stromal vascular fraction (SVF). The SVF was resuspended in NH_4_Cl, centrifuged, and filtered through a 40 μm nylon mesh to remove any cellular debris. Cells were seeded in 75 cm^2^ flasks and cultured in DMEM supplemented with 10% FBS, 100 U/mL penicillin, and 100 μg/mL streptomycin (GIBCO, Thermo Fisher Scientifics, Carlsbad, CA, USA) at 37 °C in 5% CO_2_. The characterization of adipose-derived MSCs agreed with the MSC standards established by the International Society for Cell Therapy (ISCT) [76], as we previously reported [35,77,78]

ASC-EVs were isolated from the culture medium of ASCs at 14–18 passages. ASCs were cultured to confluence, and then they were FBS deprived for 48 h to avoid the contamination of the vesicles from the serum. The culture medium of the ASCs was collected and ASC-EVs were isolated using a PureExo Exosome isolation kit (101Bio, Mountain View, CA, USA), according to the manufacturer’s protocol. The ASC-EVs were resuspended in PBS and used at different concentrations according to the experimental settings. The protein content of the ASC-EVs was determined using the Bicinchoninic Protein Assay (BCA method) according to the manufacturer’s instructions (Pierce BCATM Protein Assay, Thermo Fisher Scientifics, Carlsbad, CA, USA).

Western blot analysis was performed to verify the expression of typical ASC-EV markers, as described in Section 4.2. Ten µg of total protein from the ASCs was used as a positive control. For the ASC-EVs, a total volume of 30 µL was loaded to assess the presence of EV markers. The primary antibodies used were as follows: TSG-101 (45kDa, 1:1000 Abcam, Cambridge, UK), tetraspanin CD9 (25 kDa, 1:100, Millipore CBL-162, Burlington, MA, USA), heat shock protein 70 HSP70 (70kDa 1:100 Santa Cruz Biotechnology, Dallas, TX, USA), Alix (96kDa 1:1000, Abcam, Cambridge, UK), and GM130 (130KDa, 1:500 Thermo Fisher Scientific, Carlsbad, CA, USA). The appropriate HRP-conjugated secondary antibodies against the primary antibodies (all from Dako Agilent, Santa Clara, CA, USA) were added.

NanoSight particle-tracking analysis (NTA) was performed to detect the size and concentration of the ASC-EVs. The NanoSight NS300 system (Malvern Instruments, Malvern, UK) was configured with a scientific CMOS camera and a blue 488 nm laser was used. For the NanoSight analysis, ASC-EVs were diluted in 1 mL of PBS and injected into the instrument chamber with a 1 mL disposable syringe. The ASC-EV samples were analyzed by NTA software Version 3.4. Each of the ASC-EV samples was recorded three times for 60 s at a constant temperature of 23 °C, creating three replicable histograms, which were averaged. The camera was used at level 9, the detection threshold of the NTA software was set to 5 and the maximum jump distance, blur and the minimum track length were set to auto. The experiment was performed in triplicate.

Transmission electron microscopy (TEM) was performed to verify the morphology of the ASC-EVs. Vesicles were fixed in 2% glutaraldehyde in DNase/RNase-Free Distilled Water for 10 min on 150 mesh formvar- and carbon-coated copper grids (Societa Italiana Chimici, Rome, Italy), and dried under a hood. TEM images were acquired with a 268D Philips Morgagni TEM (Philips, Andover, MA, USA) operating at 80 kV and equipped with a Megaview II camera (Olympus Corporation, Tokyo, Japan) for digital image acquisition.

### 4.4. WST Assay

A WST assay (tetrazolium salt WST-1 (4-[3-(4-Iodophenyl)-2-(4-nitro-phenyl)-2H-5-tetrazolio]-1,3-benzene sulfonate) (Roche, Sigma Aldrich, Saint Louis, MO, USA) was used to determine the metabolic activation of SIM-A9hSOD1(G93A) and to verify the impact of ASC-EV treatment on microglial activation.

Briefly, SIM-A9hSOD1(G93A) and SIM-A9 cells were seeded into a flat bottom 96-well plate. SIM-A9 cells were used as a control. Data were obtained from three independent experiments performed in triplicate for each condition.

To verify the impact of the ASC-EV treatment on microglial activation, SIM-A9hSOD1(G93A) cells were treated with different concentrations of ASC-EVs (0.2 µg/mL, 0.4 µg/mL or 1 µg/mL) for 24 h of incubation in DMEM-F12 medium supplemented with 1% FBS. The assay was run using 10 μL of WST solution for 3 h at 37 °C following the manufacturer’s instructions. The absorbance was measured at 450 nm with a spectrophotometer (BioRad Laboratories, Milano, Italy). Data were obtained from three independent experiments performed in triplicate for each condition.

### 4.5. The Enzyme-Linked Immunospot Assay (ELISpot)

The ELISpot assay (Mabtech, Nacka Strand, Sweden) was used to assess the production of tumor necrosis factor-alpha (TNF-α) following the induction of hSOD1(G93A) expression in SIM-A9 cells by doxycycline treatment.

The assay was performed according to the manufacturer’s instructions. Briefly, microglial SIM-A9hSOD1(G93A) and SIM-A9 naïve cells were grown for 24 h. The ELISPOT plate was conditioned with DMEM/F12 containing 1% FBS + 1% FCS and incubated at room temperature for 30 min. After this period, the cells were added to the ELISPOT plate for 24 h. Subsequently, the detection antibodies were incubated, followed by color development for TNF-α spots, according to the manufacturer’s instructions.

The ELISpot analysis samples were scanned for the spot count and intensity using ELISpot Mabtech Iris-2 (Mabtech, Nacka Strand, Sweden). The TNF-α production was reported as the spot-forming units (SFU). Each SFU represented one cytokine-secreting cell producing TNF-α. The samples were run in triplicate for each test condition.

### 4.6. Morphological Characterization of SIM-A9hSOD1(G93A) Cells

To characterize the profile of microglial SIM-A9hSOD1(G93A) cells after incubation with doxycycline, the morphological parameters were evaluated. Microglial SIM-A9hSOD1(G93A) cells not treated with doxycycline were used as a control. Briefly, cells were seeded on a 24-well plate and treated with 5 μg/mL doxycycline for 24 h; after the induction of hSOD1 protein expression, they were cultured for additional 24 h and then fixed with 4% paraformaldehyde for 15 min at RT. The cells were visualized by optical microscopy (Leica DMIL, Wetlzar, Germany), and for each condition, a total of 9 random fields were acquired at 20× magnification. The images were subsequently analyzed by ImageJ v1.51j8 software to evaluate the shape of the cells by measuring the perimeter and area; the transformation index was calculated through the expression [perimeter of the cell (μm)]^2^/4π [cell area (μm^2^)], as previously described [79].

### 4.7. Immunofluorescence Staining

Immunofluorescence staining was performed on SIM-A9hSOD1(G93A) cells to label the activated cells. The cells seeded on the 24-well plate were fixed with 4% paraformaldehyde for 15 min at RT, permeabilized with 0.5% Triton for 10 min at RT, and blocked with 20% normal goat serum (Vector) or 20% donkey serum (Sigma-Aldrich) for 1 h at RT. Then, the SIM-A9hSOD1(G93A) cells were incubated overnight at 4 °C with the following primary antibodies: rabbit anti-mouse INOS (1:100; Abcam), rat anti-mouse iba1 (1:25; Abcam) and anti-His-tag (α-His) (Aviva Systems Biology, San Diego, CA, USA; 1:500). After washing with phosphate-buffered saline, appropriate fluorescein-conjugated secondary antibodies were added to the cells for 60 min at RT in the dark as follows: goat anti-rabbit IgG (1:800; Invitrogen, Thermo Fisher Scientific) conjugated with Alexa Fluor 488 nm or donkey anti-rat IgG (1:800; Invitrogen, Thermo Fisher Scientific) Alexa Fluor 594 nm, respectively. The cell nuclei were stained with DAPI (2 µg/mL, Sigma-Aldrich). The slides were mounted with Faramount Mounting Medium (Dako). The images were captured using a Zeiss Axiolab fluorescent microscope (Carl Zeiss, Oberkochen, Germany). For each condition, a total of 9 random fields were acquired at 20× magnification. The images were subsequently analyzed for the cell counts with AxioVision LE Rel. 4.5 software.

### 4.8. Flow Cytometry

Flow cytometry analysis was performed to characterize the immunophenotype of the SIM-A9hSOD1(G93A) cells after treatment with doxycycline.

The SIM-A9hSOD1(G93A) cells were cultivated in a 6-well plate and supplemented with 1% FBS in DMEM/F12 medium. Then, 1 × 10^5^ cells from each well were resuspended in 1% bovine albumin serum (BSA, AppliChem, Darmstadt, Germany) in PBS. A FIX & PERM™ Cell Permeabilization Kit (Invitrogen, Thermo Fisher Scientifics, Carlsbad, CA, USA) was used according to the manufacturer’s instructions. Then, the microglial cells were incubated with the following primary antibodies: rabbit anti-mouse INOS (1:50; Sigma Saint Louis, MO, USA) and mouse anti-mouse CD206 (1:50; Abcam, Cambridge, UK) for 1 h at RT. Afterward, the cells were incubated for 1 h at RT with the secondary antibodies: donkey anti-rabbit IgG conjugated with Alexa Fluor 647 nm (1:100; Invitrogen, Thermo Fisher Scientific, Carlsbad, CA, USA) and goat anti-mouse IgG conjugated with Alexa Fluor 488 nm (1:100; Invitrogen, Thermo Fisher Scientific, Carlsbad, CA, USA). The cell viability was assessed by incubation with 7AAD for 10 min at RT. The cell fluorescence was evaluated using the FACS BD Canto I instrument, and the data were stored and processed with FACSDiva software v 8.0 (BD, Franklin Lakes, NJ, USA). The biparametric scatter plots were analyzed with FlowJo software version vX.0.7 (Tree Star, Ashland, OR, USA).The experiment was performed in triplicate for each condition.

To verify the impact of ASC-EVs on the microglial phenotype, SIM-A9hSOD1(G93A) cells were treated with different concentrations of ASC-EVs (0.2 µg/mL, 0.4 µg/mL or 1 µg/mL) and the protocol was run as described above. All the experiments were performed in triplicate for each condition.

### 4.9. ROS Evaluation

The intracellular oxidative stress was analyzed using an ROS-sensitive probe: 5-(and-6-)- chloromethyl-2′,7′-dichlorodihydrofluorescein diacetate, acetyl ester (CM-H2DCFDA) (Thermo Fisher Scientifics, Carlsbad, CA, USA). The ROS-sensitive dye was reconstituted in DMSO (5 mM) shortly before performing the experiments and protected from light.

SIM-A9hSOD1(G93A) and SIM-A9 naïve cells were seeded on a 96-well plate and treated with 5 μg/mL of doxycycline for 24 h. For each condition, 6 replicates were performed. After treatment, the cells were washed twice with 1% BSA in PBS and loaded with 5 μM CM-H2DCFDA for 20 min at 37 °C protected from light.

The fluorescence intensity was measured using a multimode plate reader (Ex/Em 493/522 nm) (GENios Pro, Tecan, Milan, Italy) at different time points: both immediately (T0) after the incubation with the dye and after 30 (T30) and 60 min (T60). The values were normalized on SIM-A9 naïve cells fluorescence.

To evaluate the effect of ASC-EVs on ROS production by SIM-A9hSOD1(G93A) cells, after the induction of hSOD1(G93A) protein expression, they were treated with ASC-EVs at a final concentration of 1 μg/mL in DMEM-F12 supplemented with 1% FBS for 24 h and the protocol was run as described above. For each condition, 6 replicates were performed.

### 4.10. Statistical Analysis

The statistical analysis was performed using GraphPad Prism 7.00 software. Statistical significance was evaluated by Student’s T-test and one-way or two-way ANOVA, followed by Tukey’s multiple comparison test. The results were shown as the mean ± standard error of the mean (SEM). A *p* value lower than 0.05 was considered statistically significant.

## 5. Conclusions

The results presented in this paper lay the foundation for further studies in the field of ALS disease and its treatment. Future works will refine the ALS models in vitro and explore the interactions between different cell types: microglial cells, astrocytes, and motor neurons. The interplay between multiple cell types could be investigated using more complex cellular models such as co-culture systems or organoids. This will allow us to understand the complex mechanisms involved in ALS’s pathology (oxidative stress, mitochondrial dysfunction etc.) and to optimize ALS therapy with EVs in terms of the doses, time of EV administration, and safety profile.

## Figures and Tables

**Table 1:** Raw data of the size and concentration of ASC-EVs by NTA.

**Size of ASC-EV**					
**Mean (nm)**	**Mode (nm)**	**SD (nm)**	**D10 (nm)**	**D50 (nm)**	**D90 (nm)**
195.2 ± 2.2	138.7 ± 4.7	88.8 ± 6.9	127.3 ± 1.0	170.4 ± 1.7	271.9 ± 2.5
**Concentration of ASC-EV**					
**Concentration (Particles/mL)**		**Concentration (Particles/Frame)**		**Concentration (Centers/Frame)**	
4.11 × 10^9^ ± 2.03 × 10^7^		224.6 ± 1.1		201.5 ± 0.3	

## Data Availability

The data presented in this study are available on request from the corresponding author.

## Supplementary Materials

The following supporting information can be downloaded at https://www.mdpi.com/article/10.3390/ijms25115707/s1.
