# Supplementary material for: A Cellular Model of Amyotrophic Lateral Sclerosis to Study the Therapeutic Effects of Extracellular Vesicles from Adipose Mesenchymal Stem Cells on Microglial Activation
Source: Int J Mol Sci. 2024 May 24;25(11):5707. doi: 10.3390/ijms25115707 (PMC11171908; doi:10.3390/ijms25115707)
Supplement: Supplementary file 1 [file ijms-25-05707-s001.zip › ijms-2948409-supplementary.pdf]

A Cellular Model of Amyotrophic Lateral Sclerosis to Study the Therapeutic Effects of Extracellular Vesicles from Adipose Mesenchymal Stem Cells on Microglial Activation

Sylwia Dabrowska [1,2,†], Ermanna Turano [1,†], Ilaria Scambi [1], Federica Virla [1], Alice Nodari [1], Francesco Pezzini [3], Mirco Galié [1], Bruno Bonetti [4] and Raffaella Mariotti [1,*]

[1] Department of Neurosciences, Biomedicine and Movement Sciences, University of Verona, Strada Le Grazie 8, 37134 Verona, Italy; sdabrowska@imdik.pan.pl (S.D.); ermanna.turano@univr.it (E.T.); ilaria.scambi@univr.it (I.S.); federica.virla@univr.it (F.V.); alice.nodari@univr.it (A.N.); mirco.galie@univr.it (M.G.)

[2] NeuroRepair Department, Mossakowski Medical Research Institute, Polish Academy of Sciences, Pawinskiego Street 5, 02-106 Warsaw, Poland

[3] Department of Surgery, Dentistry, Paediatrics and Gynaecology (Child Neurology and Psychiatry), University of Verona, 37134 Verona, Italy; francesco.pezzini@univr.it

[4] Neurology Unit, Azienda Ospedaliera Universitaria Integrata, 37126 Verona, Italy; bruno.bonetti@univr.it

* Correspondence: raffaella.mariotti@univr.it; Tel.: +39-045-802-7164

† These authors contributed equally to this work

Supplementary Materials: The following supporting information can be downloaded at www.mdpi.com/xxx/s1.

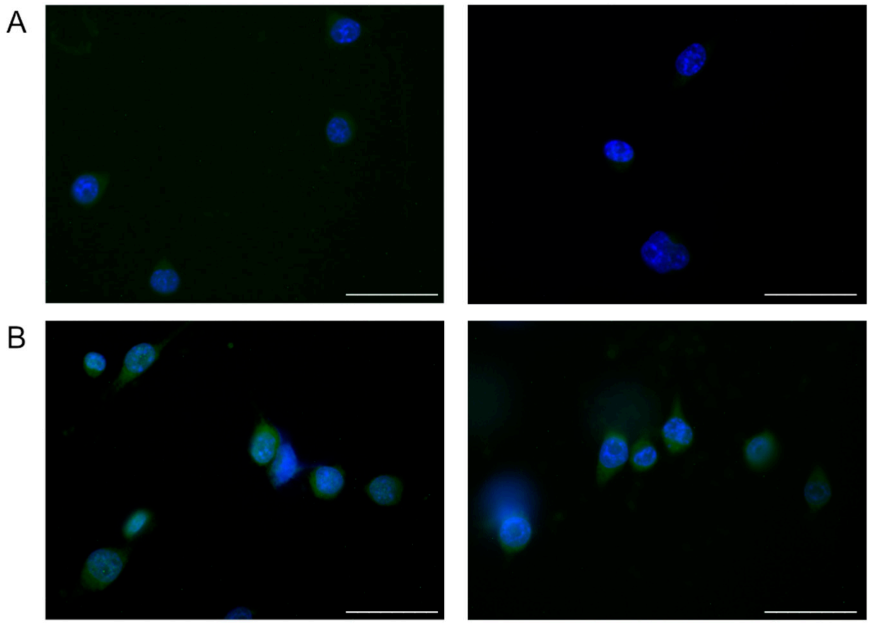

Figure S1. Detection of hSOD1(G93A)-mutated protein in SIM-A9hSOD1(G93A) microglial cells. Representative images of immunofluorescence staining with anti-His-tag (α-His) (green) to detect the presence of human SOD1-mutated protein in transiently transfected SIM-A9 cells doxycycline-treated (doxy+, (**B**)). SIM-A9hSOD1(G93A) untreated cells were used as a negative control (**A**). Nuclei were counterstained with DAPI (blue); magnification 63×, scale bar 20 μm.

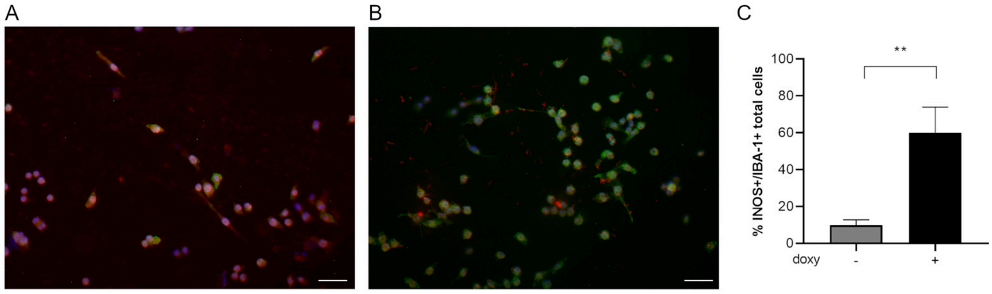

Figure S2. Evaluation of an inflammatory phenotype in SIM-A9hSOD1(G93A) microglial cells. Representative images of untreated microglial SIM-A9hSOD1(G93A) cells (**A**) and following incubation with doxycycline (doxy+) (**B**) stained with anti-iba1 antibody (red) and anti-INOS antibody (green). Nuclei were counterstained with DAPI (blue). Magnification 20×, scale bar 100 µm. (**C**) The graph shows the quantification of the INOS-positive cells, expressed as a percentage of the total number of iba1-positive SIM-A9hSOD1(G93A) microglial cells treated or untreated with doxycycline. Significant differences indicated as ** *p*<0.01 were assessed via Student's T test. Data are shown as mean ± SEM.

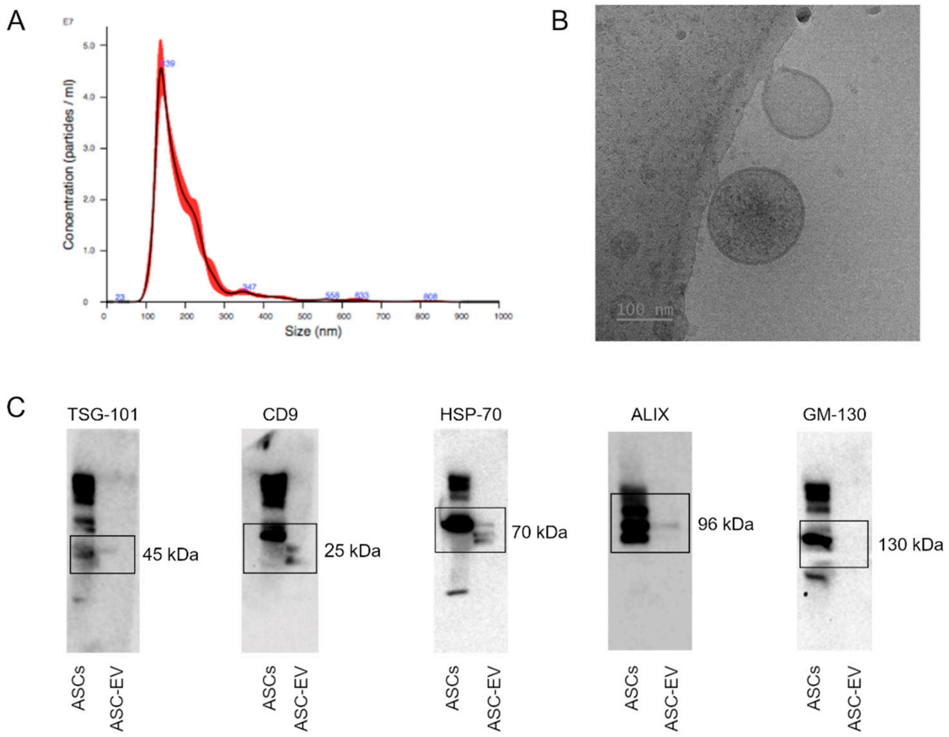

Figure S3. Characterization of ASC-EVs. (**A**) NTA graph of the concentration and particle size of the ASC-EVs. (**B**) TEM images of ASC-EVs. (**C**) Western blot of typical EV markers: TSG-101 (45 kDa), CD9 (25 kDa) HSP70 (70 kDa) and Alix (96 kDa) on ASC-EVs; GM-130 (130 kDa) was used to exclude the presence of Golgi's proteins. ASC lysates (ASCs) were used as a positive control.
